# lncRNA TINCR knockdown inhibits colon cancer cells via regulation of autophagy

**DOI:** 10.1002/fsn3.3231

**Published:** 2023-01-27

**Authors:** Jianhua Xu, Wenge Zeng, Tiantian Liu, Zhenda Wan, Xin Yang, Jun Chen, Fei Liu

**Affiliations:** ^1^ Jiangxi Province Hospital of Integrated Traditional Chinese and Western Medicine Nanchang People's Republic of China

**Keywords:** autophagy, colon cancer, HT‐29, long noncoding RNA, mTOR, SW620, TINCR ubiquitin domain containing

## Abstract

The present study aimed to evaluate the effects of long noncoding (lnc)RNA TINCR ubiquitin domain containing (TINCR) on the development of colon cancer, and the specific underlying mechanisms. The present study used adjacent healthy and cancer tissues obtained from patients with colon cancer and measured lncRNA TINCR expression using reverse transcription‐quantitative (RT‐q) PCR and in situ hybridization assays. Moreover, associations between lncRNA TINCR and clinicopathology and prognosis were also investigated. In addition, the gene and protein expression levels of lncRNA TINCR, mTOR, LC 3B, P62, and Beclin1 were measured using RT‐qPCR and western blotting assays. Cell proliferation, apoptosis, invasion, and migration were measured using MTT, Edu staining, flow cytometry, TUNEL, Transwell, and wound‐healing assays, and cell ultrastructure and LC 3B activation were measured using transmission electron microscopy and cellular immunofluorescence. Results of the present study demonstrated that lncRNA TINCR expression was significantly upregulated in colon cancer tissues, and the overall survival of the low‐expression group was significantly increased, compared with that of the high‐expression groups. In addition, the results of the present study demonstrated that lncRNA TINCR was associated with clinicopathology in patients with colon cancer. Moreover, following lncRNA TINCR knockdown using transfection with small interfering RNA‐TINCR, results of the present study demonstrated that cell proliferation was significantly reduced, while cell apoptosis was significantly increased. In addition, cell invasion and migration were significantly reduced, and autophagy was increased in HT‐29 and SW620 cell lines. However, following treatment with an mTOR agonist (an autophagy inhibitor), biological activities were significantly increased in HT‐29 and SW‐620 cell lines. Collectively, these results demonstrated that lncRNA TINCR may induce colon cancer development through the regulation of autophagy.

## Graphical abstract

lncRNA TINCR could induce colon cancer development by regulating autophagy.

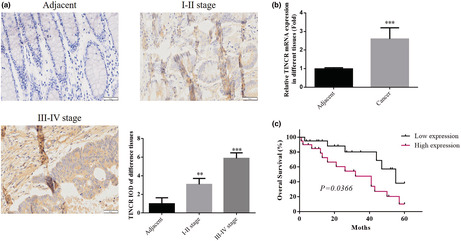

## 1 INTRODUCTION

Colorectal cancer is one of the most common malignant tumors of the digestive tract. Colon cancer is a key type of colorectal cancer and its incidence and mortality rates are increasing yearly (Chen et al., 2016). Long noncoding (lnc)RNA was first regarded as transcription ‘noise’, and initial studies demonstrated that lncRNAs possess no regulatory function. However, further investigations into lncRNAs demonstrated that they are not only involved in healthy physiological processes but also in the occurrence of diseases (Xu et al., 2019). The abnormal expression of lncRNA is present in diseases of the cardiovascular system and malignant tumors. lncRNA also acts as a potential target for the treatment of numerous diseases (Ding et al., 2018). The tissue differentiation of lncRNA induces the involvement of nonprotein coding RNA, TINCR ubiquitin domain containing (TINCR), in tumor progression. TINCR plays different roles in numerous tumor tissues; for example, it plays an inhibiting role in prostate cancer and other tumors, and plays a promotional role in non–small cell lung cancer (Dong et al., 2018; Zhu & He, 2018). Results of previous studies demonstrated that the expression levels of TINCR in gastrointestinal tumor tissues were higher than those in adjacent normal tissues (ANTs) and the high expression of TINCR is associated with the prognosis and lymphatic metastasis of patients with liver cancer (Tian et al., 2017). However, the effects of TINCR on colon cancer remain to be fully elucidated.

Notably, results of previous studies (Levy et al., 2017; Li et al., 2017, 2020; Onorati et al., 2018) demonstrated that autophagy was closely associated with the development of cancer. The interaction between autophagy and immunity in the tumor microenvironment affects both tumorigenesis and progression (Antunes et al., 2018). Moreover, results of previous studies demonstrated that lncRNA may regulate autophagy in cancer and other diseases (Guo, Wu, et al., 2019; Liang et al., 2020; Liu et al., 2018; Luo et al., 2021; Sheng et al., 2021).

Thus, the expression levels of TINCR were investigated in colon cancer tissues and ANTs in the present study. In addition, the association between TINCR expression and the pathology and prognosis of patients with colon cancer was investigated using in situ hybridization and reverse transcription‐quantitative (RT‐q) PCR. Moreover, the effects of TINCR knockdown on the bioactivity of colon cancer cells and the underlying molecular mechanisms were explored using in vitro cell experiments.

## 2 MATERIALS AND METHODS

### 2.1 Study subjects

Colon cancer tissues and corresponding ANTs (within 3 cm from the edge of the cancerous tissues) were collected from 40 patients with colon cancer, who were admitted to Jiangxi Integrated Chinese and Western Medicine Hospital from January 2018 to October 2019. These patients included 17 male and 23 female patients; 19 cases originated in the left colon and 21 cases originated in the right colon. Inclusion criteria were as follows: (i) Patients who were pathologically diagnosed with colon cancer; (ii) patients who received no local or systemic therapies (such as immunotherapy and targeted therapy) prior to the operation; and (iii) patients whose postoperative chemotherapy regimen was XELOX. Exclusion criteria were as follows: (i) Patients who exhibited complications with other tumors; and (ii) patients with immune diseases, severe liver and kidney function impairment, or infections. The present study was approved by the Ethics Committee of Jiangxi Integrated Chinese and Western Medicine Hospital. The endpoint of follow‐up visits to patients was June 10, 2020. All patients or family members signed an informed consent form. Patients were divided into low‐expression and high‐expression groups, depending on the corresponding lncRNA TINCR expression (1.00‐fold), determined using an RT‐qPCR assay.

### 2.2 Materials

Healthy colonic epithelial cells (HCoEpiC), human colon cancer cell lines HT‐29, SW620, and SW480, and LoVo and Coco‐2 cell lines were purchased from Shanghai Institute of Cell Biology. Fetal bovine serum (FBS) and DMEM/F12 were purchased from Hyclone; Cytiva. TRIzol®, Lipofectamine® 2000, RIPA lysate, and BCA protein assay kit were purchased from Invitrogen; Thermo Fisher Scientific, Inc. Pcmv plasmid, miR‐96‐5p mimics/inhibitor, and irrelevant nucleic acid sequence were purchased from Shanghai GenePharma Co., Ltd., and Annexin‐V/PI Apoptosis kit was purchased from CapitalBio Technology, Inc. Matrigel was purchased from BD Biosciences, the MTT kit was purchased from Sigma‐Aldrich; Merck KGaA, the Transwell inserts was purchased from MilliporeSigma and the dual‐luciferase detection kit was purchased from Promega Corporation. Primary antibodies against mTOR, LC 3, LC 3B, Beclin1, P62, and GAPDH were purchased from Abcam. mTOR agonist was purchased from BIOLEBO.

### 2.3 Detection of TINCR expression using ISH

To detect the expression levels of lncRNA TINCR in colon cancer tissues and ANTs, ISH was performed according to the manufacturer's instructions (Wuhan Boster Biological Technology, Ltd.). The digoxin‐labeled lncRNA TINCR probe (1:400) was added to paraffin‐embedded sections (5 μm) of tissues, and tissues were incubated at 55°C for 1 h prior to washing by PBS (5 min × 3 times) at room temperature. Subsequently, tissues were sealed with 0.2 × SSC solution at 60°C for 1 h. Following removal of the reagent, tissue sections were placed in 20% TBS‐Tween‐20 (TBST) containing an antidigoxin antibody (cat no. ab30512, Abcam) (1:200) and incubated at 37°C for 1 h. Tissues were stained using H&E and observed and photographed under the optical microscope (CX23, OLYMPUS).

### 2.4 RT‐qPCR

Total RNA was extracted from cells or tissues using TRIzol® reagent. RNA was reverse transcribed into cDNA according to the instructions of the Reverse Transcription kit (Thermo Fisher Scientific, Inc.). qPCR was performed using SYBR Mixture (Takara Biotechnology Co., Ltd.), and the times and temperatures were determined using the preexperiment. Thermocycling conditions for qPCR were as follows: Initial denaturation at 95°C for 10 min following predenaturation; followed by 40 cycles of denaturation at 95°C for 5 s, annealing at 60°C for 30 s and extension at 72°C for 32 s. Primers used for RT‐qPCR are displayed in Table 1. mRNA levels were quantified using the 2^−ΔΔCt^ method and normalized to the internal reference gene, GAPDH. Experimental procedures were repeated independently three times.

**TABLE 1:** Primer sequence.

Gene name	F:(5′‐3′)	R:(5′‐3′)
TINCR	TGTGGCCCAAACTCAGGGATACAT	AGATGACAGTGGCTGGAGTTGTCA
LC 3B	GTCGACATGCCGTCGGAGAAGACC	GGATCCCACTGACAATTTCATCCCGA
mTOR	GGCTTCTGAAGATGCTGTCC	GAGTTCGAAGGGCAAGAGTG
Beclin1	CACATCTGGGACAGTGGACAGT	GCATGGAGCAGCAACACAGTCT
P62	TCCAGCAGAGGCACAGAAGACAAGAG	CAGTCATCGTATCCTCCTGAGCAGTT
GAPDH	GGTGAAGGTCGGTGTGAACG	GCTCCTGGAAGATGGTGATGG

### 2.5 Cell culture and transfection

HT‐29 and SW620 cells were cultured with DMEM/F12 containing 10% FBS in an incubator with 5% CO_2_ at 37°C. When cell density reached 70%–80%, 0.25% trypsin solution was used for digestion. Cells were centrifuged at 310 × g and ambient temperature at room temperature for 5 min, and the supernatant was subsequently discarded. PBS was used for resuspension and adjustment of cell density to 5 × 10^5^/ml. A quantity of 1 ml cell suspension was seeded in a 6‐well culture plate and incubated as previously described. When cells were fully attached, the medium was removed for transfection. Small interfering RNA (si)‐NC (cat. no. 12935200; Thermo Fisher Scientific, Inc.) or si‐TINCR (cat. no. AM16708; Thermo Fisher Scientific, Inc.) were transfected into colon cancer cells at a final concentration of 50 nmoL/L, according to the instructions for Lipofectamine® 2000 reagent. mTOR was dissolved in dimethyl sulfoxide (DMSO) to reach a final concentration of 5 mg/L, and cells were incubated as previously described. Following 4‐h incubation at room temperature, the culture solution was replaced with DMEM/F12 containing 15% FBS. Effects of transfection were observed under a fluorescence microscope, photographed, and recorded. Cells were cultured in an incubator for 48 h for subsequent experiments at room temperature.

### 2.6 MTT detection

Cells in all groups were treated with different treatments for 48 h at room temperature, and cell density was adjusted to 5 × 10^3^ cells/ml. Cells were seeded in a 96‐well microplate. Each well contained 200 μl DMEM/F12 culture solution with 10% FBS. Cells were cultured in an incubator with 5% CO_2_ at 37°C. Following 48 h, 20 μl MTT (final concentration, 5 mg/m1) was added to each well, and the mixture was incubated at 37°C for 4 h. Subsequently, the original culture solution was discarded, and 150‐μl DMSO was added to each well. The mixture was agitated at room temperature for 10 min to completely dissolve the purple formazan crystals formed.

The absorbance at 450 nm was decided using a microplate reader (Thermo Fisher Scientific, Inc.).

### 2.7 EdU staining

Cells in all groups were treated with different treatments for 48 h at room temperature, and 10 μmoL/L EdU reagent was added to cells, according to the instructions for the EdU fluorescent staining cell proliferation kit. Cells were incubated for 2 h at room temperature. EdU that did not infiltrate into DNA was washed out with PBS, and cells were fixed in 4% paraformaldehyde for 30 min at room temperature. Subsequently, the fixative was removed using PBS. Following washing, 2 mg/ml apollo stain was added and cells were incubated at room temperature in a dark place for 30 min. Stain was removed using PBS, and DAPI was used for nuclear staining. Five fields were randomly selected and observed under an IX73 fluorescence microscope. ImageJ software v1.46 (National Institutes of Health) was used to count EdU‐positive cells.

### 2.8 Flow cytometry (FCM) assay

Cells in all groups were treated with different treatments for 48 h at room temperature and 0.25% trypsin solution was used for routine digestion. Cells were washed three times with PBS, and subsequently centrifuged at 800 g × min for 5 min at 4°C. The supernatant was discarded and cell concentration was adjusted to 5 × 10^5^ cells in each sample. A total of 195 μl Annexin V‐FITC combined with buffer solution was added to resuspend the cells. Subsequently, 5 Ixl Annexin V, FITC, and 10 μl propidium iodide (PI) were added, and cells were incubated at room temperature in a dark place for 30 min. Analysis was performed using a FACScan flow cytometer with CellQuest software version 5.1 (BD Biosciences). These experimental procedures were repeated three times.

### 2.9 TUNEL staining

Cells in all groups were treated with different treatments for 48 h, and fixed with 4% paraformaldehyde at a normal temperature of 37 °C for 30 min, followed by the cultivation with TUNEL solution for 1 h at 37°C. The cells were then stained with 3,3‐diaminobenzidine (Sigma‐Aldrich; Merck KGaA) for 10 min at room temperature according to the manufacturer's protocol. Cell nuclei were stained with 0.1 μg/ml DAPI for 5 min at room temperature and nuclear DNA fragmentation was assessed using the DeadEnd™ Fluorometric TUNEL system (Promega Corporation). Finally, the cells were observed in five randomly selected fields under an Olympus IX71 fluorescence microscope (Olympus Corporation). TUNEL‐positive cells and total cells were analyzed using ImageJ 1.8.0 software (National Institutes of Health). Red fluorescence was indicative of TUNEL‐positive cells.

### 2.10 Transwell assay

Cell invasion was evaluated using 8‐mm pore Transwell inserts (Costar; Corning, Inc.) which were precoated with Matrigel (BD Biosciences) at 37°C for 2 h. Briefly, cells were dissociated into single cells and resuspended in DMEM at a density of 1 × 10^5^ cells/ml. A quantity of 200 μl cell suspension was added to the upper chamber, and 800 μl DMEM supplemented with 10% FBS was added to the lower chamber. Following incubation for 24 h at 37°C, cells on the upper surface of the upper chamber were removed, and cells on the lower surface of the upper chamber were fixed in 4% paraformaldehyde at room temperature for 10 min. Cells were subsequently stained with 0.25% crystal violet (MilliporeSigma) at room temperature for 10 min, followed by three washes with PBS. Images of stained cells from five random views were captured under an X71 (U‐RFL‐T) fluorescence microscope (Olympus Corporation; magnification, ×20).

### 2.11 Wound‐healing assay

Cells in all groups were treated with different treatments for 48 h, and following trypsin digestion and centrifugation at room temperature for 24 h, cells were resuspended with a complete medium. A cell counter was used to adjust the cell concentration to 2 × 10^5^ cells/well. Cells were seeded in a six‐well microplate at 2 ml/well and subsequently observed under a microscope. When the cell density reached 80–90%, the medium in the microplate was discarded. A wound was created using a pipette tip, and cells were subsequently washed twice with PBS. The residual cells were removed. Changes in migration between 0 and 48 h were observed and photographed under an inverted microscope. The average wound‐healing rate was analyzed and calculated.

### 2.12 Western blot analysis

Cells in all groups were treated with different treatments for 48 h, collected, and centrifuged at room temperature for 24 h to discard the supernatant. Cells were lysed with cell lysates, after which the supernatant was collected. The protein content in the supernatant was quantified using BCA solution. Following 12% SDS‐PAGE gel electrophoresis, proteins (60 μg/per lane) in the gel were transferred to PVDF membranes and blocked with 5% skimmed milk powder for 2 h at 4°C. Following blocking, the membrane was washed five times with 0.2% TBST for 10 min each time, And then incubated with primary antibodies against mTOR (1:500; cat. no. ab134903), LC 3 (1:500; cat. no. ab221794), Beclin1 (1:500, cat. no. ab302669), P62 (1:500, cat. no.ab140651), and GAPDH (1:500; cat. no. ab8245) at room temperature for 3 h, and then incubated with horseradish peroxidase‐conjugated goat anti‐rabbit secondary antibody (1:5000; cat. no. ab6721) at room temperature for 1 h. All antibodies were purchased from Abcam. Following secondary incubation, membranes were washed five times with TBST for 10 min each time, and protein bands were visualized using ECL luminous fluid. Following visualization with the gel imaging system, the gray value of each band was calculated, and GAPDH was used as the loading control.

### 2.13 LC 3B protein expression detection using immunofluorescent staining

Cells in all groups were treated with different treatments for 48 h, the culture plate was removed and the supernatant was discarded. Cells were washed with PBS, and fixed in 4% paraformaldehyde at room temperature for 15 min. The membrane was incubated with 0.1% TritonX‐100 for 10 min and subsequently blocked with 5% BSA at room temperature for 1 h. The membrane was incubated with anti‐LC 3B (1:100, cat. no. ab239416, Abcam) antibody at 4°C overnight. The slides were further incubated with Alex 488‐labeled secondary antibodies for 1 h in a moist chamber at room temperature and washed again. Topro‐3 (diluted 1:100,000 in PBS) was added for nuclear staining and incubated for 10 min, the slides were washed with PBS, and anti‐fade mounting medium was added, then covered with a cover slip, sealed with nail polish, and examined by fluorescence microscopy. Micrographs were taken for result assessment. Following nuclear staining, the amount of LC 3B imported into the nucleus of cells in each group was observed under a ZeissAxiovert200M inverted microscope (Zeiss GmbH).

### 2.14 Ultrastructural observation of tumor cells in each group using transmission electron microscopy (TEM)

Cells in all groups were treated with different treatments for 48 h, and fixed in 4% paraformaldehyde for 2 h. Cells were subsequently washed using PBS, fixed with 1% osmic acid for 2 h, and washed again using PBS. Cells were dehydrated using gradient ethanol and acetone. A 1‐μm semi‐thin section was stained with toluidine blue, and an ultrathin section was stained with lead citrate and uranyl acetate. Sections were observed using a transmission electron microscope and photographed for comparative analysis.

### 2.15 Statistical analysis

Data are presented as the mean ± standard deviation, and SPSS 22.0 (IBM Corp.) was used for statistical analysis. Unpaired data were analyzed using Student's t‐tests, and counting data were analyzed using a χ^2^ test and expressed as a percentage. *p* < .05 was considered to indicate a statistically significant difference.

## 3 RESULTS

### 3.1 Association between lncRNA TINCR expression and the prognosis of patients with colorectal cancer

**FIGURE 1:**
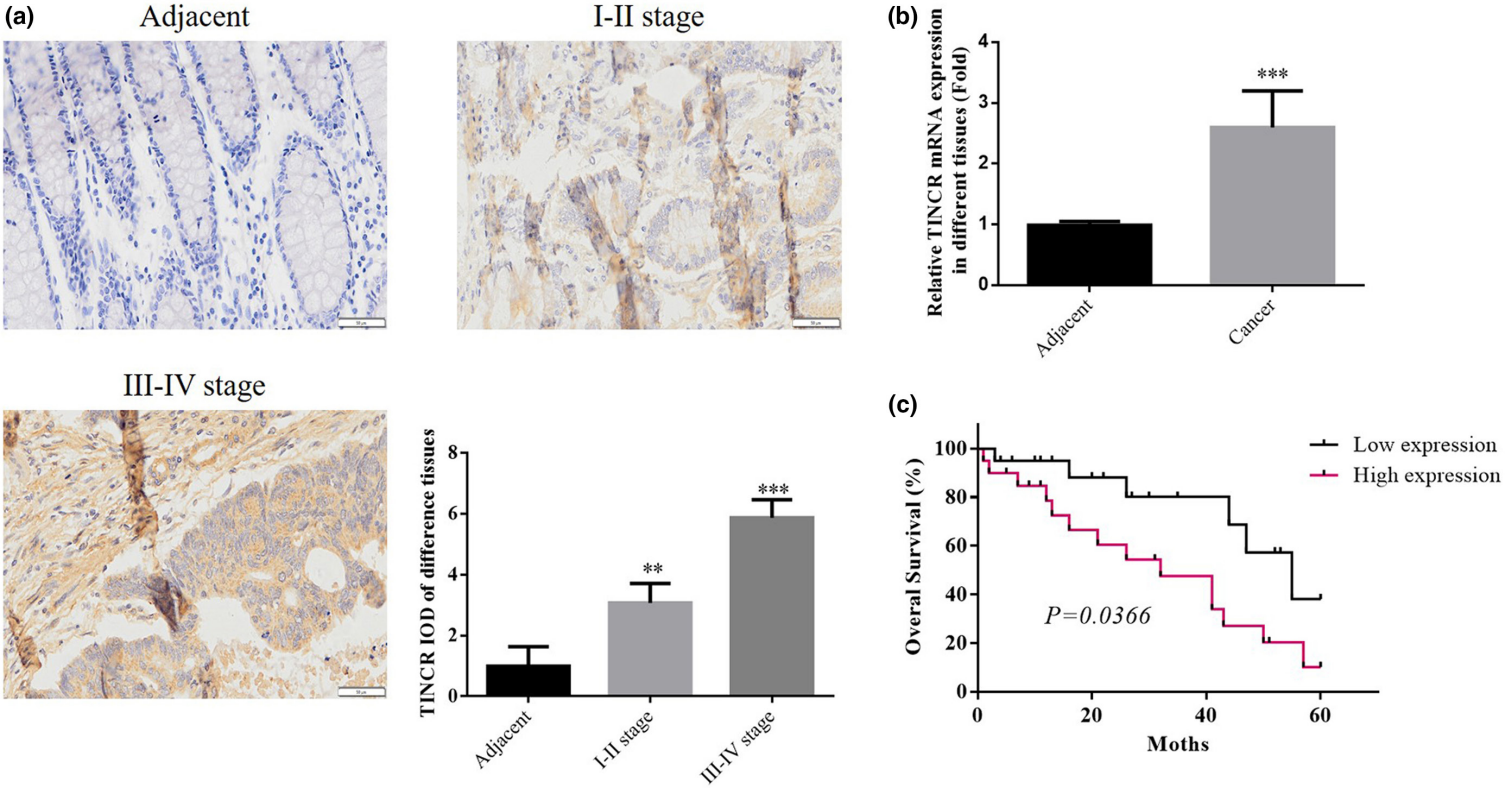
lncRNA TINCR expression in colon cancer and ANTs and its relation with the prognosis of patients with colorectal cancer. (a) lncRNA TINCR expression in different tissues by ISH assay (200×). (b) lncRNA TINCR expression in different tissues by qRT‐PCR assay. (c) Analysis of correlation between lncRNA TINCR and prognosis. **p < .01, ***p < .001, compared with adjacent.

Results of the present study demonstrated that when compared with ANTs (Figure 1a), the expression levels of lncRNA TINCR were significantly elevated in tissues obtained from patients with stage I–II and III–IV colon cancer (*p* < 0.01). Results of the RT‐qPCR analysis demonstrated that compared with ANTs, the gene expression levels of lncRNA TINCR were significantly elevated in colon cancer tissues (*p* < 0.001; Figure 1b). Patients with colon cancer were divided into low‐ and high‐expression groups, according to the median (2.88‐fold of lncRNA expression in colon cancer tissues). Results of the present study demonstrated that the overall survival of patients in the low‐expression group was significantly higher than that in the high‐expression group (*p* = 0.0366; Figure 1c).

### 3.2 Association between lncRNA TINCR expression levels and clinicopathologic features of patients with colon cancer

Results of the present study demonstrated that the expression levels of lncRNA TINCR were independent of patients' age, sex, tumor location, T, or N staging (*p* > 0.05); however, lncRNA TINCR expression levels were associated with M and clinical staging (*p* < 0.05). The expression levels of lncRNA TINCR in patients with stages M1 and III–IV colon cancer were higher than those in patients with stages M0 and I–II colon cancer (*p* < 0.05; Table 2).

**TABLE 2:** Relationship between lncRNA TINCR expression and the clinical features of colon cancer patients (*n*, %).

Clinical feature	Low expression (*n* = 20)	High expression (*m* = 20)	*t*	*p*
Age (year)	58.6 ± 11.3	59.3 ± 13.5	0.851	.844
Gender			0.101	.734
Male	12 (60.0)	11 (55.0)		
Female	8 (40.0)	9 (45.0)		
Location			1.604	.205
Left side	12 (60.0)	7 (35.0)		
Right side	8 (40.0)	13 (65.0)		
T stage			0.573	.449
T1 + T2	6 (30.0)	3 (15.0)		
T3 + T4	14 (70.0)	17 (85.0)		
N stage			1.823	.177
N0	9 (45.0)	4 (20.0)		
N1 + N2	11 (55.0)	16 (80.0)		
M stage			5.104	.024
M0	16 (80.0)	8 (40.0)		
M1	4 (20.0)	12 (60.0)		
Clinical stage			10.800	.001
I–II	10 (50.0)	0.00		
III–IV	10 (50.0)	20 (100)		

### 3.3 Effects of lncRNA TINCR knockdown on the proliferation of colon cancer cells

**FIGURE 2:**
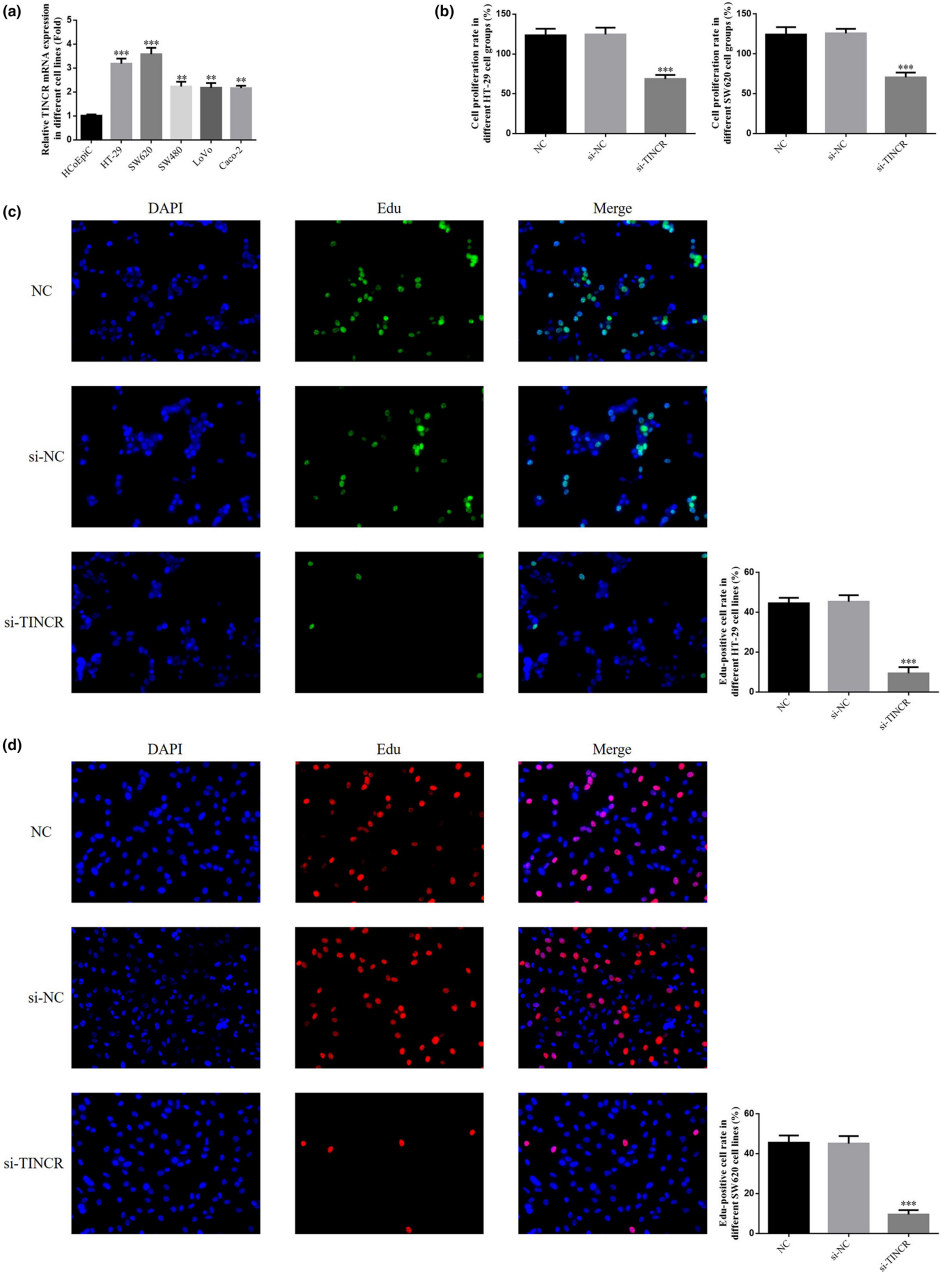
lncRNA TINCR expression in different cells and its knockout on the proliferation of colon cancer cells. NC: The cells were treated with normal; si‐NC: The cells were transfected with negative control; si‐TINCR: The cells were transfected with si‐TINCR which knockdown lncRNA TINCR expression. (a) lncRNA TINCR gene expression in different cell lines by qRT‐PCR. **p < .01, ***p < .001, compared with HCoEpiC. (b) Cell proliferation in different cell lines by MTT assay. ***p < 0.001, compared with NC group. (c) Edu‐positive cell rate in different HT‐29 cell lines (200×). ***p < 0.001, compared with NC group. (d) Edu‐positive cell rate in different SW620 cell lines (200×). ***p < 0.001, compared with NC group.

Compared with HCoEpiC, the gene expression levels of lncRNA TINCR in HT‐29, SW620, SW480, LoVo, and Coco‐2 colon cancer cells were significantly elevated (*p* < 0.01; Figure 2a). Notably, the expression levels of lncRNA TINCR were highest in HT‐29 and SW620 cells. Therefore, HT‐29 and SW620 cells were selected for subsequent analysis. Results of the MTT assay demonstrated that following lncRNA TINCR knockdown, the proliferation rate of both HT‐29 and SW620 cells in the si‐TINCR group was significantly decreased, compared with the NC group (*p* < 0.001; Figure 2b). Results of the EdU staining demonstrated that the number of EdU‐positive cells was significantly lower in HT‐29 and SW620 cells, compared with the NC group (*p* < 0.001; Figure 2c,d).

### 3.4 lncRNA TINCR knockdown promotes cell apoptosis in colon cancer cell lines

**FIGURE 3:**
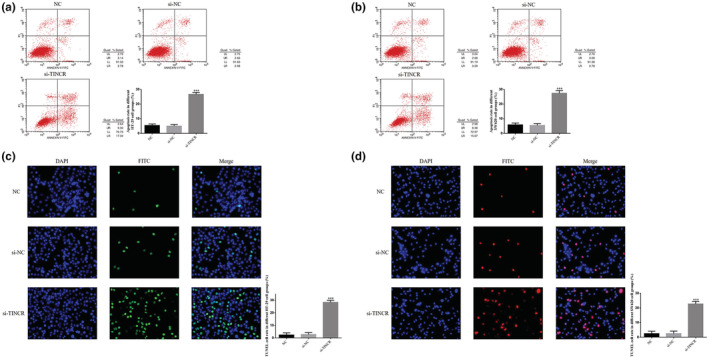
lncRNA TINCR knockdown promotes cell apoptosis in colon cancer cell lines. NC: The cells were treated with normal; si‐NC: The cells were transfected with negative control; si‐TINCR: The cells were transfected with si‐TINCR which knockdown lncRNA TINCR. (a) Apoptosis rate in different HT‐29 cell groups (%). (b) Apoptosis rate in different SW620 cell groups (%). (c) TUNEL cell rate in different HT‐29 cell groups (%). (d) TUNEL cell rate in different SW620 cell groups (%). ***p < .001, compared with NC group.

FCM detection demonstrated that the apoptosis rate of HT‐29 and SW620 cells in the si‐TINCR group was significantly increased, following lncRNA TINCR knockdown (*p* < 0.001; Figure 3a,b). Moreover, TUNEL staining demonstrated that the number of apoptotic cells in the si‐TINCR groups was significantly increased, compared with the NC groups (*p* < 0.001; Figure 3c,d).

### 3.5 lncRNA TINCR knockdown suppresses colon cancer cell invasion and migration

**FIGURE 4:**
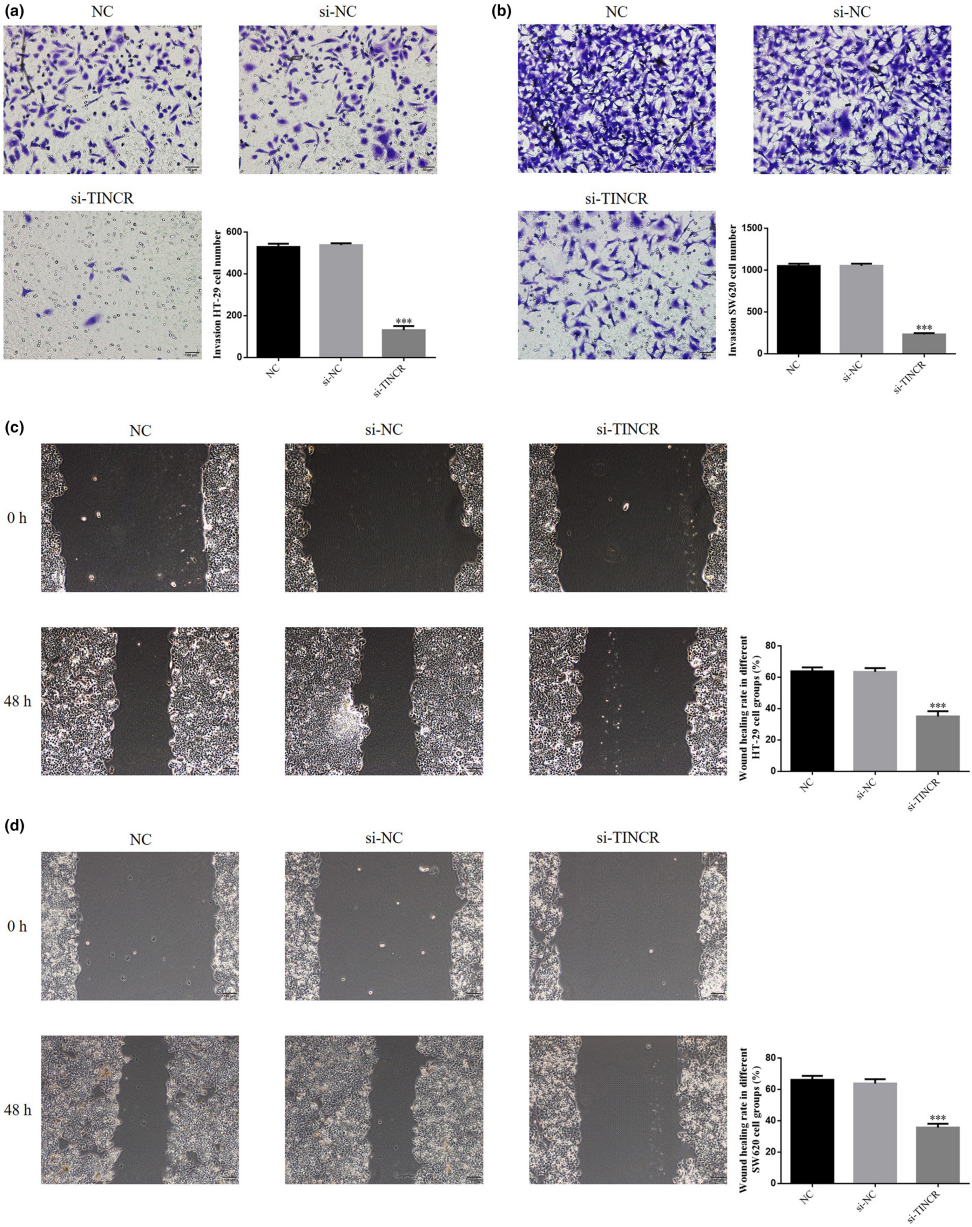
lncRNA TINCR knockdown suppresses colon cancer cell invasion and migration. NC: The cells were treated with normal; si‐NC: The cells were transfected with negative control; si‐TINCR: The cells were transfected with si‐TINCR which knockdown lncRNA TINCR. (a) Invasion HT‐29 cell number (200×). (b) Invasion SW620 cell number (200×). (c) Wound‐healing rate in different HT‐29 cell groups (200×). (d) Wound‐healing rate in different SW620 cell groups (200×). ***p < .001, compared with NC group.

Results of the Transwell assay demonstrated that the number of invading HT‐29 and SW620 cells in the si‐TINCR groups significantly decreased, compared with the NC groups (*p* < 0.001; Figure 4a,b). Results of the wound‐healing analysis demonstrated that the wound‐healing rate of HT‐29 and SW620 cells in the si‐TINCR groups was significantly reduced, compared with the NC groups (*p* < 0.001; Figure 4c,d).

### 3.6 lncRNA TINCR knockdown impacts cell ultrastructure

**FIGURE 5:**
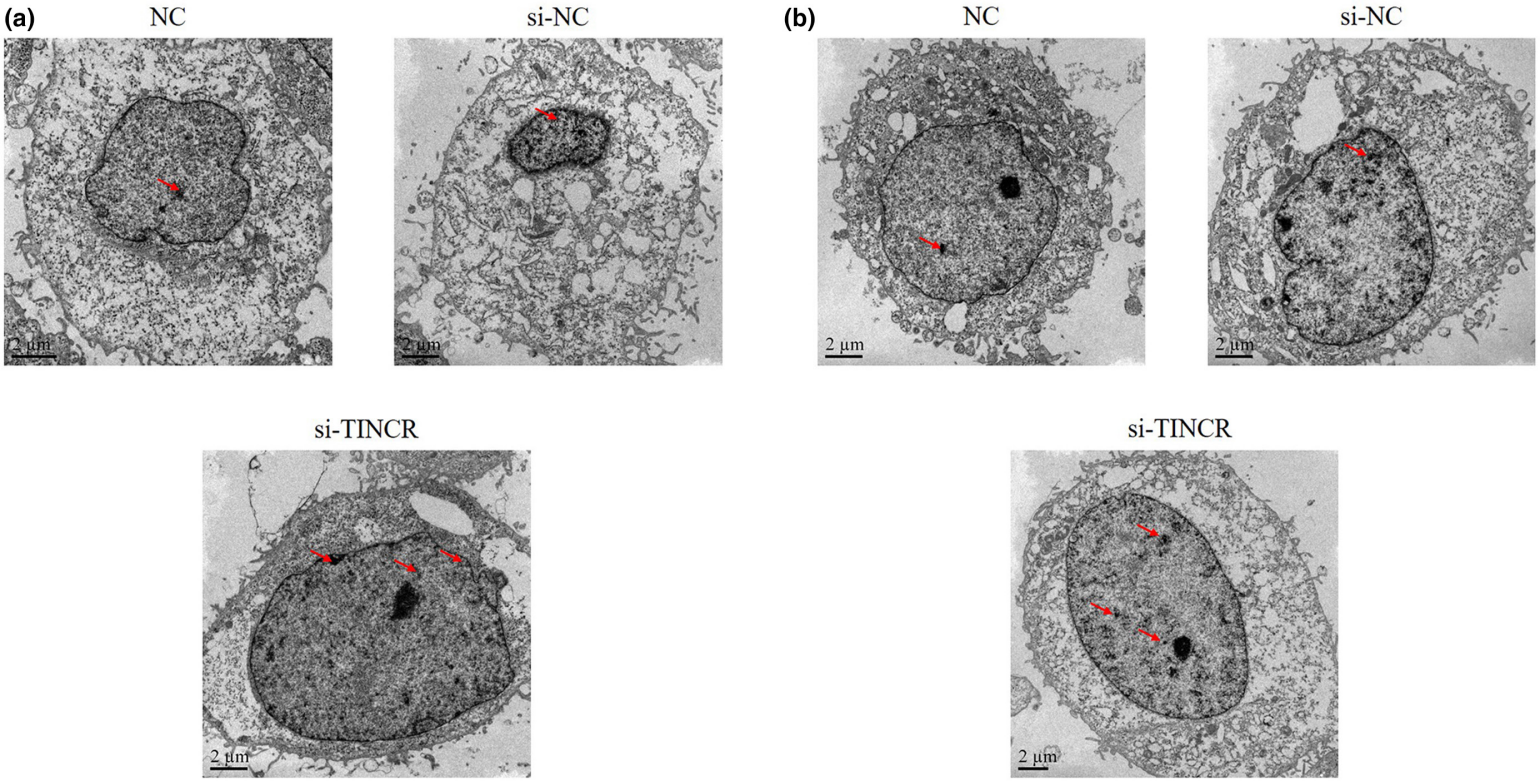
LncRNA TINCR knockdown affects cell ultrastructure (5000×). NC: The cells were treated with normal; si‐NC: The cells were transfected with negative control; si‐TINCR: The cells were transfected with si‐TINCR which knockdown lncRNA TINCR (Autophagosomes are shown as red arrow).

Results of TEM demonstrated that the ultrastructure of HT‐29 and SW620 cells was healthy in NC and si‐NC groups. Notably, there were some autophagosomes in HT‐29 and SW620 cells in the si‐TINCR groups (Figure 5a,b).

### 3.7 lncRNA TINCR knockdown impacts gene and protein expression

**FIGURE 6:**
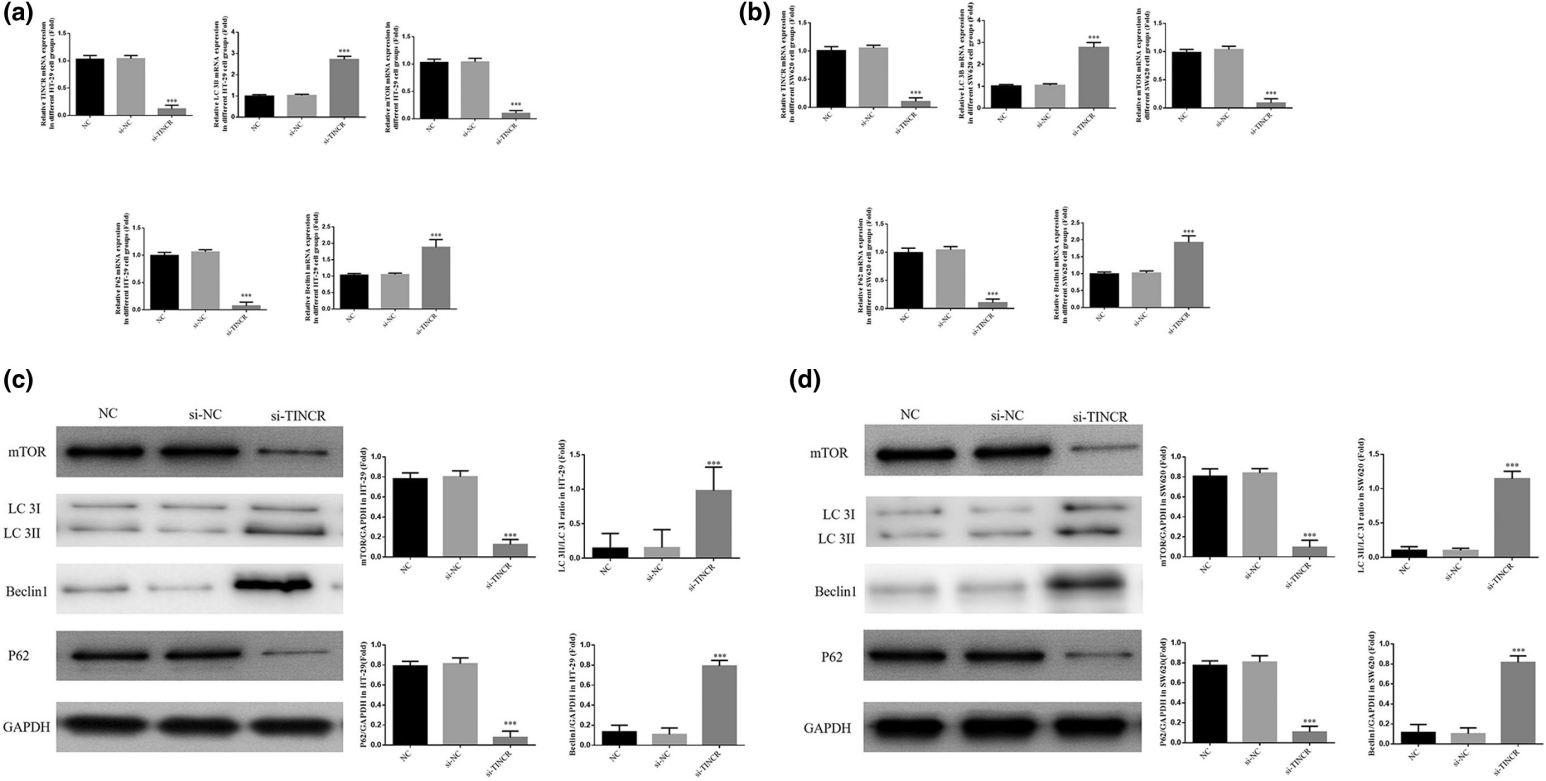
lncRNA TINCR knockdown had the effect on relative gene and protein expression. NC: The cells were treated with normal; si‐NC: The cells were transfected with negative control; si‐TINCR: The cells were transfected with si‐TINCR which knockdown lncRNA TINCR. (a) lncRNA TINCR knockdown had the effect on relative gene expression in HT‐29 cell groups. (b) lncRNA TINCR knockdown had the effect on relative gene expression in SW620 cell groups. (c) lncRNA TINCR knockdown had the effect on relative protein expression in HT‐29 cell groups by WB assay. (d) lncRNA TINCR knockdown had the effect on relative protein expression in SW620 cell groups by WB assay. ***p < .001, compared with NC group.

Results of the RT‐qPCR analysis demonstrated that the gene expression levels of lncRNA TINCR, mTOR, and P62 were significantly decreased, and Beclin1 and LC 3B were significantly increased in the si‐TINCR groups, compared with the NC group in HT‐29 and SW620 cell lines (*p* < 0.001; Figure 6a,b). In addition, results of the western blotting analysis demonstrated that the protein expression levels of mTOR and P62 proteins were significantly decreased, and Beclin1 and LC 3II/LC 3I ratios were significantly increased in the si‐TINCR groups, compared with the NC groups (*p* < 0.001; Figure 6c,d).

### 3.8 lncRNA TINCR knockdown impacts LC 3B expression

**FIGURE 7:**
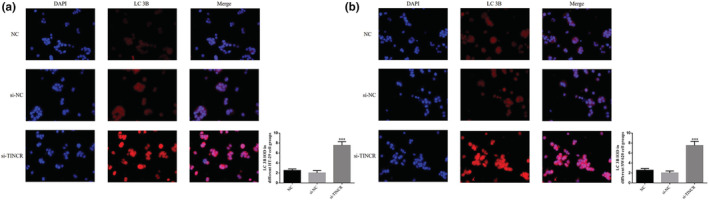
lncRNA TINCR knockdown had the effect on LC 3B protein expression. NC: The cells were treated with normal; si‐NC: The cells were transfected with negative control; si‐TINCR: The cells were transfected with si‐TINCR which knockdown lncRNA TINCR. (a) LC 3B nuclear volume in different HT‐29 cell groups (200×). (b) LC 3B nuclear volume in different SW620 cell groups (200×). ***p < .001, compared with NC group.

Cell immunofluorescence detection demonstrated that the amount of LC 3B protein expression in HT‐29 and SW620 cells in the si‐TINCR group was significantly increased, compared with the NC groups (*p* < 0.001; Figure 7a,b).

### 3.9 Treatment with an mTOR agonist (autophagy inhibitor) inhibits the proliferation of colon cancer cells following TINCR knockdown

**FIGURE 8:**
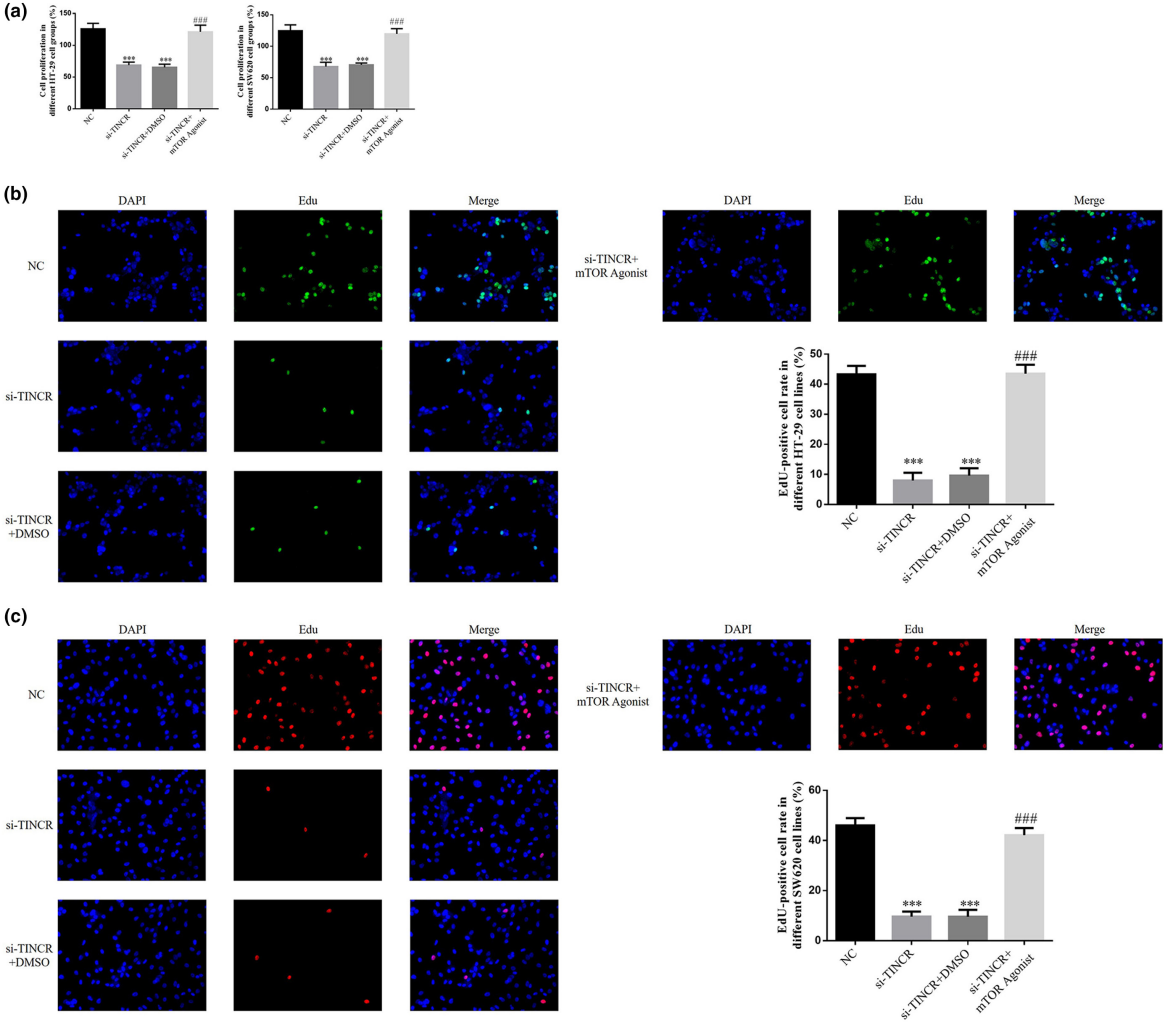
Effect of mTOR agonist (autophagy inhibitor) in inhibition of proliferation of colon cancer cells by knockout of TINCR. NC: The cells were treated with normal; si‐TINCR: The cells were transfected with si‐TINCR which knockdown lncRNA TINCR; si‐TINCR+DMSO: The cells were transfected with si‐TINCR which knockdown lncRNA TINCR and transfected with DMSO; si‐TINCR+mTOR agonist: The cells were transfected with si‐TINCR which knockdown lncRNA TINCR and treated with mTOR agonist. (a) The cell proliferation rate by MTT assay. (b) Edu‐positive cell rate in different HT‐29 cell lines (200×). (c) Edu‐positive cell rate in different SW620 cell lines (200×). ***p < 0.001, compared with NC group; ^###^ *p* < 0.001, compared with si‐TINCR group.

Results of the MTT analysis demonstrated that the proliferation rate of HT‐29 and SW620 cells in the si‐TINCR and si‐TINCR + DMSO groups was significantly lower than that in the NC group (*p* < 0.001; Figure 8a). Following treatment with the mTOR agonist, the proliferation rate of HT‐29 and SW620 cells in the si‐TINCR + mTOR agonist groups was notably higher than that in the si‐TINCR group (*p* < 0.001; Figure 8a). In addition, results of the EdU staining analysis demonstrated that the number of EdU‐positive HT‐29 and SW620 cells in the si‐TINCR group was significantly reduced, compared with the NC group (*p* < 0.001; Figure 8b,c). Moreover, following treatment with the mTOR agonist, the number of Edu‐positive HT‐29 and SW620 cells in the si‐TINCR + mTOR agonist group was notably higher than that in the si‐TINCR group (*p* < 0.001; Figure 8b,c).

### 3.10 Treatment with an mTOR agonist (autophagy inhibitor) promotes the apoptosis of colon cancer cells following TINCR knockdown

**FIGURE 9:**
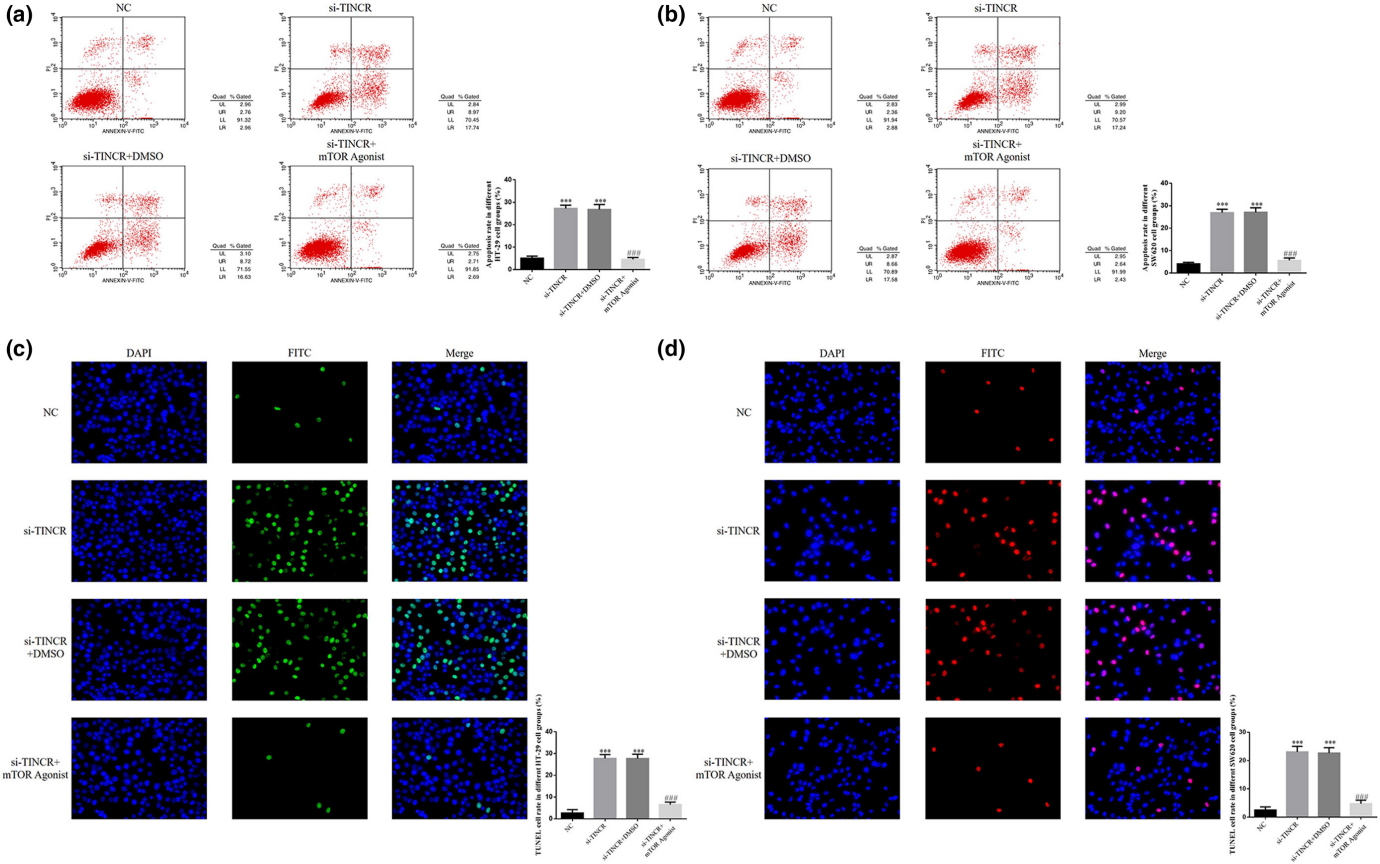
Effect of mTOR agonist (autophagy inhibitor) in promotion of apoptosis of colon cancer cells by knockout of TINCR. NC: The cells were treated with normal; si‐TINCR: The cells were transfected with si‐TINCR which knockdown lncRNA TINCR; si‐TINCR+DMSO: The cells were transfected with si‐TINCR which knockdown lncRNA TINCR and transfected with DMSO; si‐TINCR+mTOR agonist: The cells were transfected with si‐TINCR which knockdown lncRNA TINCR and treated with mTOR agonist. (a) Apoptosis rate in different HT‐29 cell groups. (b) Apoptosis rate in different SW620 cell groups. (c) TUNEL cell rate in different HT‐29 cell groups. (d) TUNEL cell rate in different SW620 cell groups. ***p < .001, compared with NC group; ^###^ *p* < .001, compared with si‐TINCR group.

Results of the FCM analysis demonstrated that the apoptosis rate of HT‐29 and SW620 cells in the si‐TINCR and si‐TINCR + DMSO groups was significantly higher than that in the NC group (*p* < 0.001; Figure 9a,b). Following treatment with the mTOR agonist, the apoptosis rate of HT‐29 and SW620 cells in the si‐TINCR + mTOR agonist group was notably reduced, compared with the si‐TINCR group (*p* < 0.001; Figure 9a,b). Results of the TUNEL detection analysis demonstrated that the rate of positive apoptotic HT‐29 and SW620 cells in the si‐TINCR and si‐TINCR+DMSO groups was significantly higher than that in the NC group (*p* < 0.001; Figure 9c,d). In addition, following treatment with the mTOR agonist, the positive apoptotic cell rate in the si‐TINCR + mTOR agonist group was notably reduced, compared with HT‐29 and SW620 cells in the si‐TINCR group (*p* < 0.001; Figure 9c,d).

### 3.11 Treatment with an mTOR agonist (autophagy inhibitor) inhibits the invasion and migration of colon cancer cells following TINCR knockdown

**FIGURE 10:**
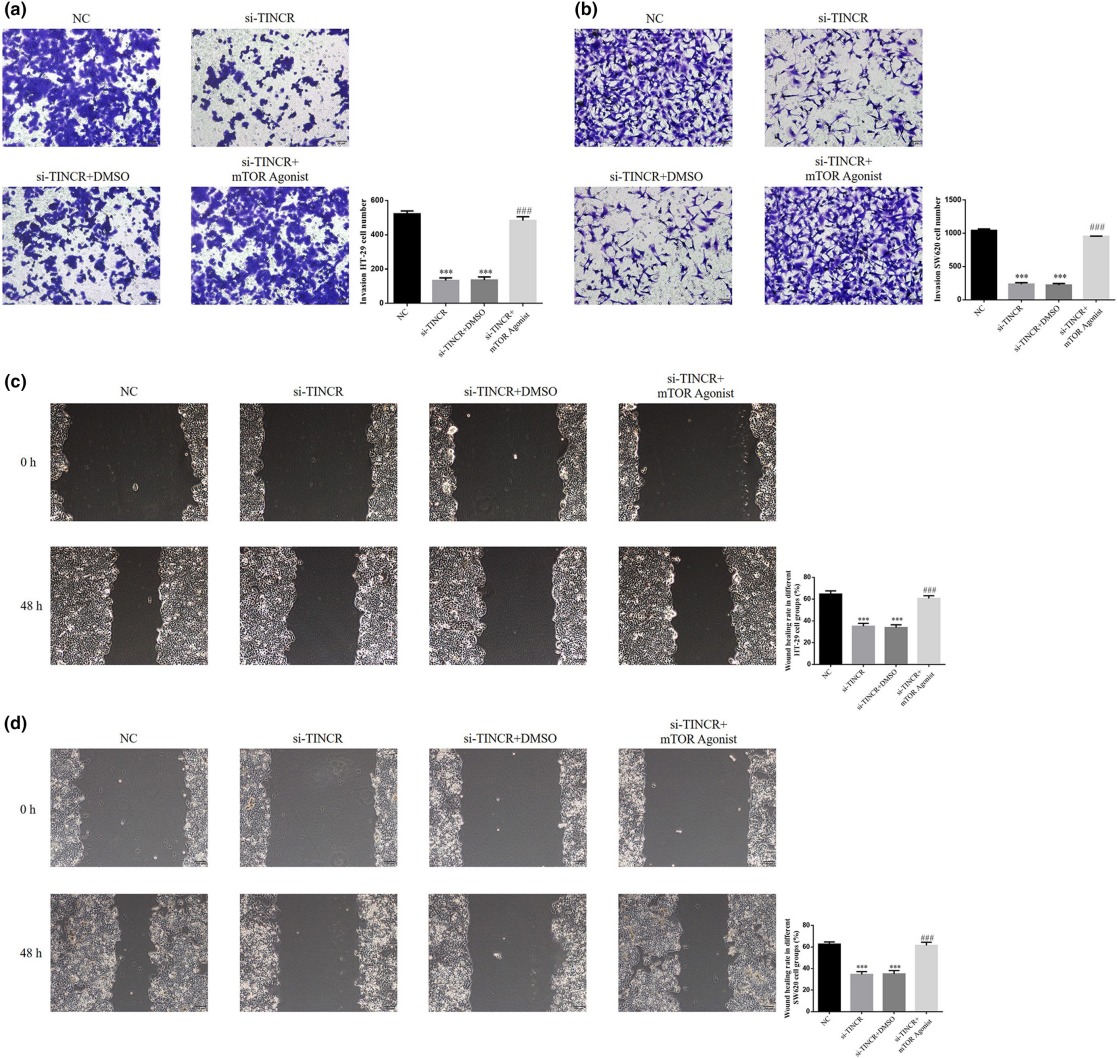
Effect of mTOR agonist (autophagy inhibitor) in inhibition of invasion and migration of colon cancer cells by knockout of TINCR. NC: The cells were treated with normal; si‐TINCR: The cells were transfected with si‐TINCR which knockdown lncRNA TINCR; si‐TINCR+DMSO: The cells were transfected with si‐TINCR which knockdown lncRNA TINCR and transfected with DMSO; si‐TINCR+mTOR agonist: The cells were transfected with si‐TINCR which knockdown lncRNA TINCR and treated with mTOR agonist. (a) Invasion HT‐29 cell number (200×). (b) Invasion SW620 cell number (200×). (c) Wound‐healing rate in different HT‐29 cell groups (100×). (d) Wound‐healing rate in different SW620 cell groups (100×). ***p < .001, compared with NC group; ^###^ *p* < .001, compared with si‐TINCR group.

Results of the Transwell analysis demonstrated that the number of invading HT‐29 and SW620 cells in the si‐TINCR and si‐TINCR + DMSO groups was significantly reduced, compared with the NC group (*p* < 0.001; Figure 10a,b). Following treatment with the mTOR agonist, the number of invading HT‐29 and SW620 cells in the si‐TINCR + mTOR agonist group was notably higher than that in the si‐TINCR group (*p* < 0.001; Figure 10a,b). Moreover, the wound‐healing rate of HT‐29 and SW620 cells in the si‐TINCR and si‐TINCR + DMSO groups was significantly lower than that in the NC group (*p* < 0.001; Figure 10c,d). Following treatment with the mTOR agonist, the wound‐healing rate of HT‐29 and SW620 cells in the si‐TINCR + mTOR agonist group was notably higher than that in the si‐TINCR group (*p* < 0.001; Figure 10c,d).

### 3.12 Treatment with an mTOR agonist (autophagy inhibitor) impacts the ultrastructure of colon cancer cells following TINCR knockdown

**FIGURE 11:**
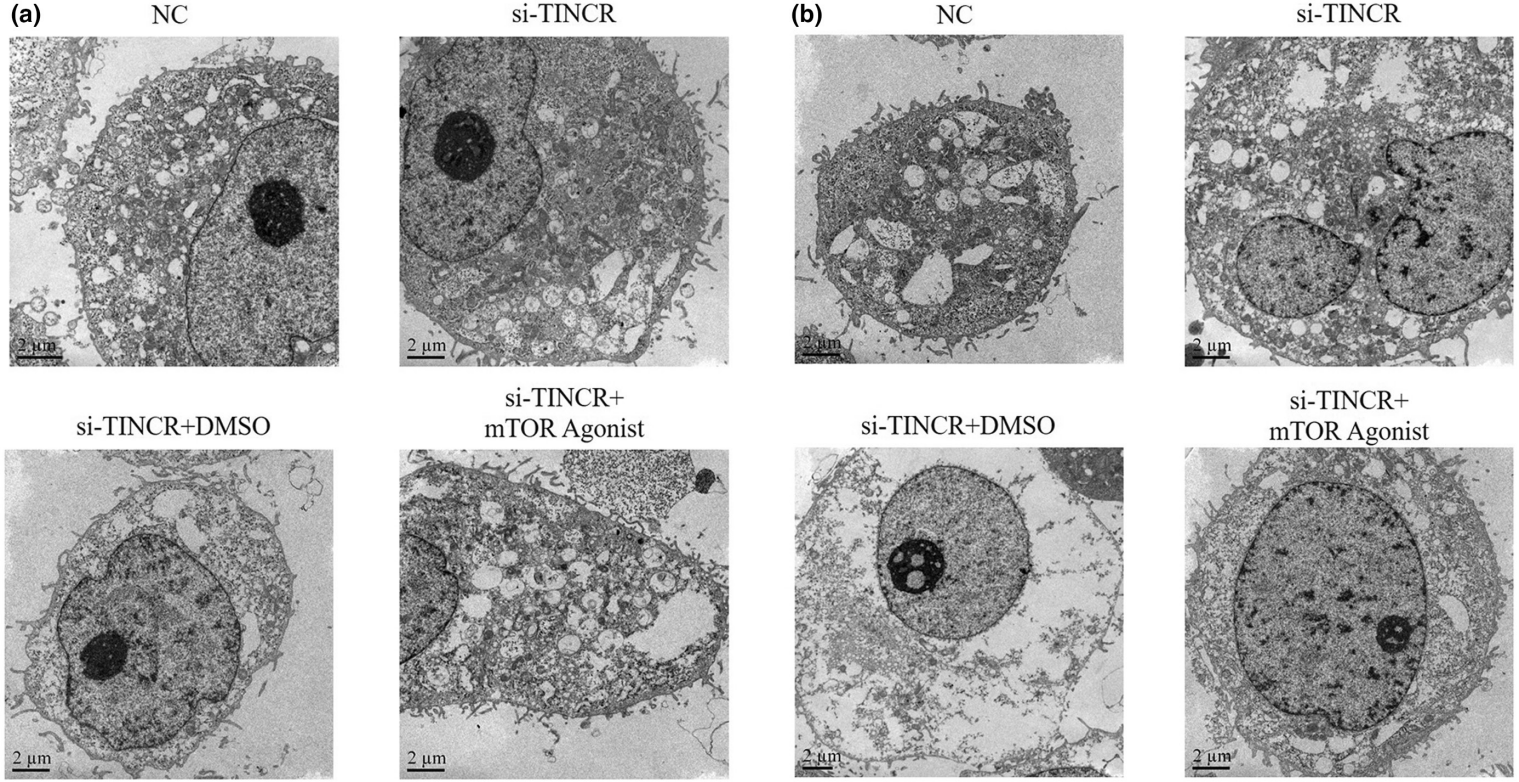
Effect of mTOR agonist (autophagy inhibitor) in ultrastructure of colon cancer cells by knockout of TINCR. NC: The cells were treated with normal; si‐TINCR: The cells were transfected with si‐TINCR which knockdown lncRNA TINCR; si‐TINCR+DMSO: The cells were transfected with si‐TINCR which knockdown lncRNA TINCR and transfected with DMSO; si‐TINCR+mTOR agonist: The cells were transfected with si‐TINCR which knockdown lncRNA TINCR and treated with mTOR agonist (Autophagosomes are shown as red arrow).

Results of the TEM analysis demonstrated that there were few autophagosomes in the NC group. However, following lncRNA TINCR knockdown, an increased number of autophagosomes was observed in HT‐29 and SW620 cells. Following treatment with the mTOR agonist, the number of autophagosomes was markedly reduced (Figure 11a,b).

### 3.13 Relative gene and protein expression

**FIGURE 12:**
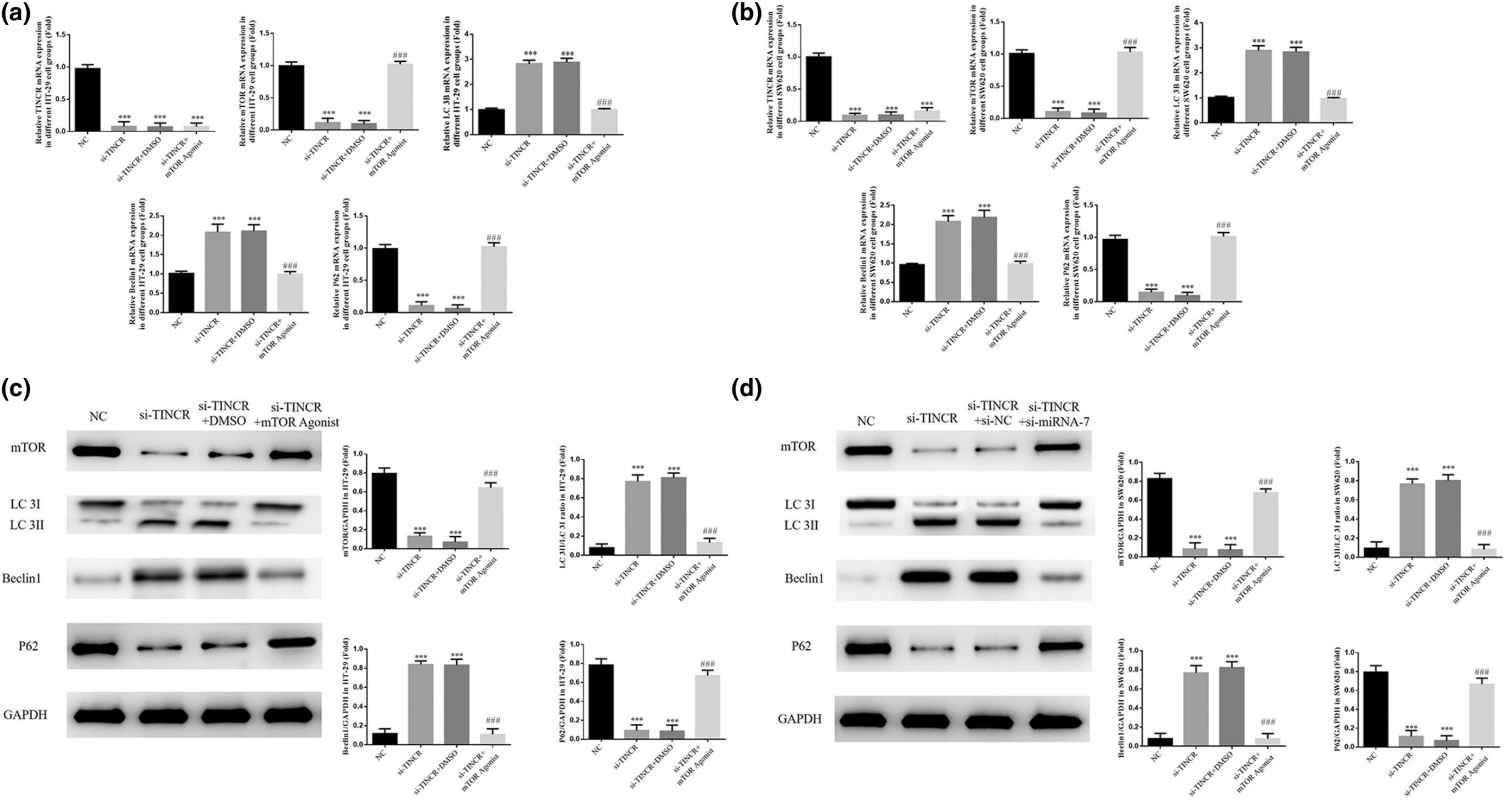
Relative gene and protein expression. NC: The cells were treated with normal; si‐TINCR: The cells were transfected with si‐TINCR which knockdown lncRNA TINCR; si‐TINCR+DMSO: The cells were transfected with si‐TINCR which knockdown lncRNA TINCR and transfected with DMSO; si‐TINCR+mTOR agonist: The cells were transfected with si‐TINCR which knockdown lncRNA TINCR and treated with mTOR agonist. (a) Relative gene expression in HT‐29 cell groups. (b) Relative gene expression in SW620 cell groups. (c) Relative protein expression in HT‐29 cell groups. (d) Relative protein expression in SW620 cell groups. ***p < .001, compared with NC group; ^###^ *p* < .001, compared with si‐TINCR group.

Results of the RT‐qPCR analysis demonstrated that the gene expression levels of TINCR in HT‐29 and SW620 cells in the si‐TINCR, si‐TINCR + DMSO, and si‐TINCR + mTOR agonist groups were significantly decreased (*p* < 0.001; Figure 12a,b). In addition, the gene expression levels of LC 3B and Beclin1 in the si‐TINCR and si‐TINCR+DMSO groups were significantly increased (*p* < 0.001; Figure 12a,b), and the gene expression levels of mTOR and P62 were significantly decreased (*p* < 0.001; Figure 12a,b). Following treatment with the mTOR agonist, the gene expression levels of LC 3B and Beclin1 in the si‐TINCR + mTOR agonist group were significantly decreased, compared with the si‐TINCR group. Moreover, the gene expression levels of mTOR and P62 were significantly increased (*p* < 0.001; Figure 12a,b). Results of the western blot analysis demonstrated that the protein expression levels of mTOR and P62 in the si‐TINCR and si‐TINCR + DMSO groups were significantly reduced, and the ratio of LC 3II/LC 3I and levels of Beclin1 protein expression were significantly higher than those in the NC group (*p* < 0.001; Figure 12c,d). Following treatment with the mTOR agonist, the protein expression levels of mTOR and P62 were markedly increased, and the ratio of LC 3II/LC 3I and levels of Beclin1 protein expression were significantly lower in the si‐TINCR + mTOR agonist group than those in the si‐TINCR group (*p* < 0.001; Figure 12c,d).

### 3.14 LC 3B expression in each group

**FIGURE 13:**
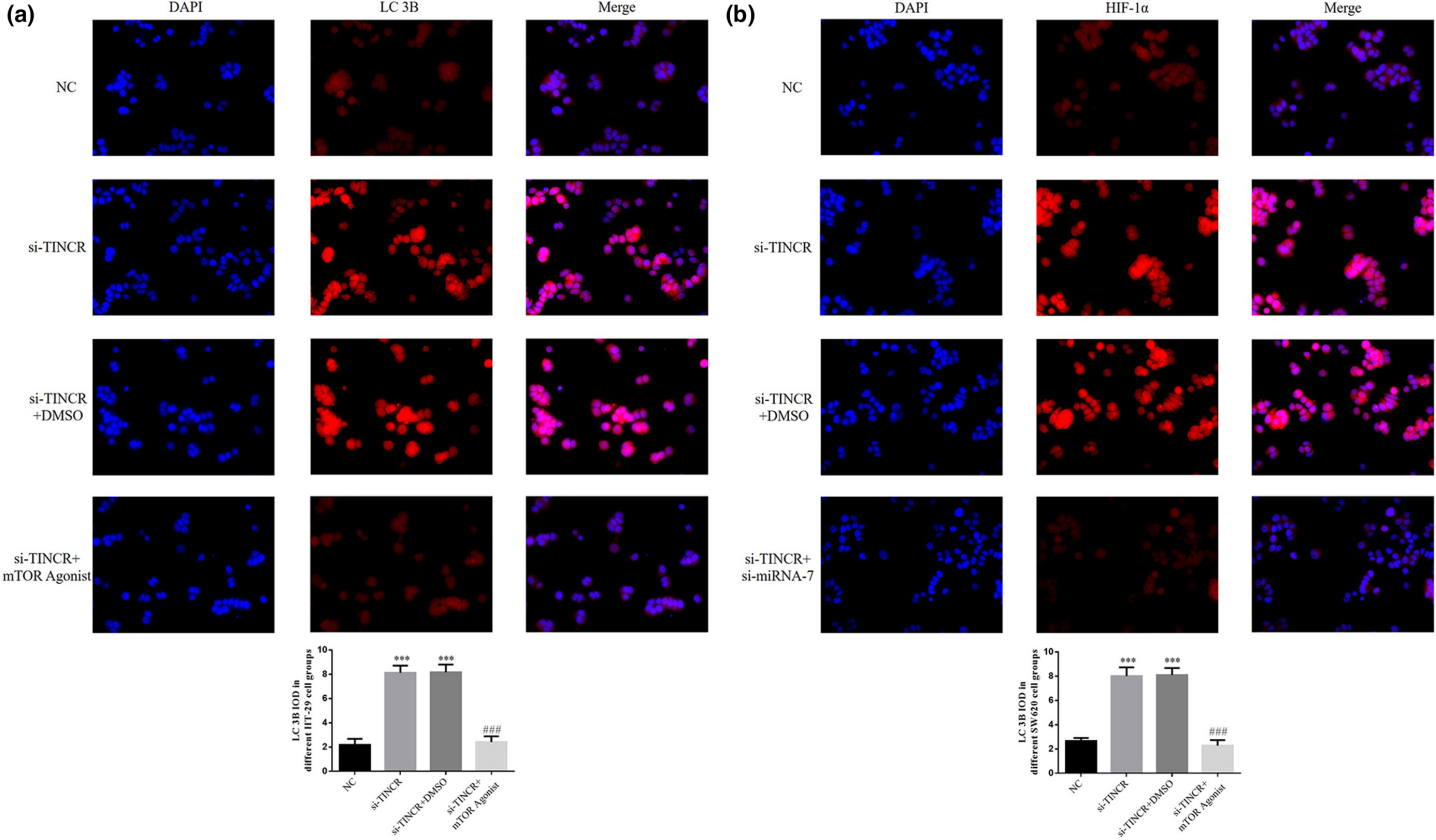
Detection of the amount of LC 3B protein expression. NC: The cells were treated with normal; si‐TINCR: The cells were transfected with si‐TINCR which knockdown lncRNA TINCR; si‐TINCR+DMSO: The cells were transfected with si‐TINCR which knockdown lncRNA TINCR and transfected with DMSO; si‐TINCR+mTOR agonist: The cells were transfected with si‐TINCR which knockdown lncRNA TINCR and treated with mTOR agonist. (a) LC 3B IOD in different HT‐29 cell groups (200×). (b) LC 3B IOD in different SW620 cell groups (200×). ***p < .001, compared with NC group; ^###^ *p* < .001, compared with si‐TINCR group.

Results of the immunofluorescence detection analysis demonstrated that the amount of LC 3B imported in HT‐29 and SW620 cells in the si‐TINCR and si‐TINCR+DMSO groups was significantly higher than that in the NC group (*p* < 0.001; Figure 13a,b). Following treatment with the mTOR agonist, the amount of LC 3B imported in HT‐29 and SW620 cells in the si‐TINCR + mTOR agonist group was notably lower than that in the si‐TINCR group (*p* < 0.001; Figure 13a,b).

## 4 DISCUSSION

The majority of genomes in eukaryotes are transcribed. During transcription, numerous functions of lncRNAs are generated, prior to translation. lncRNA is mainly formed by the transcription of RNA polymerase II and has numerous functions, including regulating DNA methylation, chromatin remodeling, protein modification, and affecting gene transcription. lncRNA possesses multiple modes of action and regulatory mechanism, and plays different roles in numerous physiological processes of the human body (Zhang et al., 2018). TINCR promotes cell proliferation and is involved in tumorigenesis in a number of tumors (Dong et al., 2018; Tian et al., 2017). TINCR expression is upregulated in lung cancer, and upregulation is associated with the clinicopathological features of patients with tumors. Therefore, the malignant metastatic potential of tumor cells may be inhibited through downregulation of TINCR expression (Zhu & He, 2018). TINCR acts as a tumor suppressor in prostate cancer and inhibits tumor metastasis (Dong et al., 2018). Moreover, TINCR is overexpressed in liver cancer, highlighting that TINCR may act as a promoter of liver cancer progression (Tian et al., 2017).

Results of the present study demonstrated that the expression levels of lncRNA TINCR in colon cancer tissues are higher than those in ANTs, indicating that lncRNA TINCR may play a role in promoting cancer. Further clinical analysis demonstrated that the expression of lncRNA TINCR in stage M1 patients is higher than that in stage M0 patients and that its expression level in patients with stage III–IV colon cancer is also higher than that in patients with stage I–II colon cancer. These results indicated that the high expression of lncRNA TINCR may be relevant to tumor progression. Notably, the higher the expression quantity, the more likely the tumor is to metastasize. Moreover, the results of the present study demonstrated that lncRNA TINCR is overexpressed in colon cancer cell lines. Thus, we hypothesized that lncRNA TINCR may be an oncogenic gene in colon cancer. In addition, following lncRNA TINCR knockdown in vitro, results of the present study demonstrated that the bioactivity (proliferation, invasion, and migration) of HT‐29 and SW620 cells was significantly inhibited, indicating that TINCR may play a promoting role in the progression of colon cancer. Moreover, lncRNA TINCR knockdown may effectively inhibit the tumorigenesis and progression of colon cancer.

Results of previous studies demonstrated that lncRNA regulates cancer development via autophagy (Li et al., 2021; Yang et al., 2019). Results of the present study demonstrated that TINCR knockdown impacted the biological activities of colon cancer cells. On the other hand, autophagy was significantly increased. However, following treatment with an mTOR agonist, the antitumor effects induced by lncRNA TINCR knockdown were reversed. Thus, we hypothesized that the antitumor effects of lncRNA TINCR knockdown are closely associated with autophagy.

Autophagy is unique to eukaryotes. To maintain the stability of the intracellular environment, autophagy is present in the occurrence, development, and treatment of tumors (Glick et al., 2010). Autophagy is strictly regulated by autophagy‐related genes (Parzych & Klionsky, 2014), such as Beclin1, which is also an important tumor suppressor gene (Zhu et al., 2021). Results of a previous study demonstrated that the expression levels of Beclin‐1 in tumor cells were significantly reduced, and upregulation of these expression levels may play a role in tumor inhibition (Xu & Qin, 2019). Moreover, P62 plays a major role in the regulation of autophagy and is involved in the occurrence and development of tumors (Jiang et al., 2015). Tao et al. (2020) demonstrated that regulation of P62 expression inhibited the growth of lung cancer (Tao et al., 2020). LC 3 is one of the markers of autophagy, participating in the formation of the autophagic membrane and in the regulation of binding of lysosomes and autophagosomes (Tanida et al., 2008). When autophagy occurs, the activated LC 3 I is modified to form LC 3 II. Following the transfer of the fusion protein, LC 3II promotes autophagy maturation on the autophagy membrane (Runwal et al., 2019). The ratio of LC 3 II to LC 3 I is often used as a standard to measure the degree of autophagy (Fan et al., 2021). Results of a previous study demonstrated that following the induction of autophagy in cancer cells, the protein expression ratio of LC 3 II/LC 3I in tumor cells was significantly increased (Guo, Pei, et al., 2019). In addition, results of previous studies demonstrated that reduced mTOR expression may induce autophagy (Kim & Guan, 2015; Wang & Zhang, 2019). Notably, the results of the present study demonstrated that lncRNA TINCR knockdown exhibited antitumor effects to reduce mTOR gene and protein expression, and this may be associated with autophagy in colon cancer. Moreover, the results of the present study also demonstrated that the antitumor effects induced by lncRNA TINCR knockdown were reversed following treatment with an mTOR agonist, and autophagy was inhibited.

## 5 CONCLUSION

lncRNAs play important parts in some diseases including oncology; however, lncRNA TINCR's effects and mechanism in colon cancer remain unclear. Abnormally high‐expression levels of lncRNA TINCR play an important role in the tumorigenesis and progression of colon cancer. In addition, the mechanisms of action of lncRNA TINCR may be closely associated with the mTOR‐induced regulation of autophagy in vitro.

## COMPLIANCE WITH ETHICAL STANDARDS SECTION

This study was approved by the Ethics Committee of Jiangxi Integrated Chinese and Western Medicine Hospital.

## COMPLIANCE WITH ETHICAL STANDARDS

There was no conflict of interest in this study.

## Data Availability

The data that support the findings of this study are available on request from the corresponding author.
